# When Cannabis Use Goes Wrong: Mental Health Side Effects of Cannabis Use That Present to Emergency Services

**DOI:** 10.3389/fpsyt.2021.640222

**Published:** 2021-02-15

**Authors:** Candice E. Crocker, Alix J. E. Carter, Jason G. Emsley, Kirk Magee, Paul Atkinson, Philip G. Tibbo

**Affiliations:** ^1^Department of Psychiatry, Dalhousie University, Halifax, NS, Canada; ^2^Department of Diagnostic Radiology, Dalhousie University, Halifax, NS, Canada; ^3^Department of Emergency Medicine, Dalhousie University, Halifax, NS, Canada; ^4^Emergency Health Services, Halifax, NS, Canada; ^5^Horizon Health Network, Saint John, NB, Canada

**Keywords:** cannabis, adverse events, cannabis induced psychosis, acute intoxication, mental health, emergency department, emergency transport

## Abstract

Cannabis use is a modifiable risk factor for the development and exacerbation of mental illness. The strongest evidence of risk is for the development of a psychotic disorder, associated with early and consistent use in youth and young adults. Cannabis-related mental health adverse events precipitating Emergency Department (ED) or Emergency Medical Services presentations can include anxiety, suicidal thoughts, psychotic or attenuated psychotic symptoms, and can account for 25–30% of cannabis-related ED visits. Up to 50% of patients with cannabis-related psychotic symptoms presenting to the ED requiring hospitalization will go on to develop schizophrenia. With the legalization of cannabis in various jurisdiction and the subsequent emerging focus of research in this area, our understanding of who (e.g., age groups and risk factors) are presenting with cannabis-related adverse mental health events in an emergency situation is starting to become clearer. However, for years we have heard in popular culture that cannabis use is less harmful or no more harmful than alcohol use; however, this does not appear to be the case for everyone. It is evident that these ED presentations should be considered another aspect of potentially harmful outcomes that need to be included in knowledge mobilization. In the absence of a clear understanding of the risk factors for mental health adverse events with cannabis use it can be instructive to examine what characteristics are seen with new presentations of mental illness both in emergency departments (ED) and early intervention services for mental illness. In this narrative review, we will discuss what is currently known about cannabis-related mental illness presentations to the ED, discussing risk variables and outcomes both prior to and after legalization, including our experiences following cannabis legalization in Canada. We will also discuss what is known about cannabis-related ED adverse events based on gender or biological sex. We also touch on the differences in magnitude between the impact of alcohol and cannabis on emergency mental health services to fairly present the differences in service demand with the understanding that these two recreational substances may impact different populations of individuals at risk for adverse events.

## Introduction

Cannabis is one of the most frequently used recreational drugs in the world with the United Nations Office on Drugs and Crime estimating that 192 million of the global population used in the past year (1). Cannabis-related adverse events, such as those requiring presentation to an emergency department (ED) or Emergency Medical Services (EMS) presentations, have had limited research compared to some of the other potential longer term negative effects, and the limited research and knowledge translation in this whole area has not fully addressed the public perception that cannabis use is harmless, being as safe or safer than alcohol (2–4). However, there is a growing body of evidence showing that like all other drugs known to mankind, some individuals will indeed experience adverse outcomes with cannabis use.

Cannabis use is becoming recognized as a modifiable risk factor for several adverse effects on human health, including mental illness (5). While the literature indicates a strong association with the development of psychotic disorders; mood and anxiety disorders as well as suicidal ideation have also been reported (5–7). Although physical health is not the focus of this article, there are several reported medical adverse events that are of concern, such as cannabinoid hyperemesis syndrome, lung injury with vaping cannabis and arrhythmias (8–11). Additionally, the role of cannabis in trauma (e.g., motor vehicle collisions), injuries (e.g., falls), and in acute negative effects in conjunction with illicit drug use, are causes of ED admissions (12).

With respect to cannabis use as a modifiable risk factor for the development and exacerbation of mental illness, there are signals emerging from ongoing research that indicate that early (e.g., adolescent) and regular (daily or almost daily) use, as well as the use of high potency products [high in delta-9-tetrahydrocannabinol (THC)] may be particular risk variables (13–15). These risk factors appear to decrease the average age for developing a mental illness and are attributed to an increased incidence of mental illness and increase the risk for development of a cannabis use disorder [e.g., for psychosis (14, 16)]. Cannabis use is also associated with exacerbation of and possibly development of anxiety disorders and depressive disorders but the evidence is mixed and not yet as extensive as that for the association with psychosis (7, 17, 18). When gender is considered, women tend to use less cannabis, but what minimal evidence exists suggests that women may be at even greater risk of negative effects; further, data outside a binary gender spectrum with cannabis use are almost non-existent (19). It remains unclear why some individuals develop these conditions as an adverse reaction to cannabis use while others do not. Genetic factors are likely involved and research focusing on this interaction has been promising; however, work to date has suggested that most mental illness is polygenic in origin and thus our understanding of the genetic basis for both acute and long term adverse effects may take some time to unravel (20–22). Another avenue of research in this area is the study of epigenetic mechanisms (e.g., DNA methylation) which has also shown some promise (23). Ultimately, modifying cannabis risk behaviors and early identification of high risk individuals may be our best approach from a public health standpoint in reducing both acute and long term adverse events.

Early identification and treatment of illness is vital to maximize positive outcomes in both physical and mental health. Early intervention services (EIS) for mental illness have been shown to significantly alter disease trajectory, decreasing personal, family and health care burden (24). However, a significant number of index (first) referrals to mental health care are from the Emergency Department (ED), implying that mental health concerns have already reached a critical level such that emergency services are required. For example, between 50 and 55% of youth and young adults accepted to EIS for psychosis are being referred from ED pathways (19, 25). Importantly, there is also a significantly high level of cannabis use in the EIS for psychosis demographic (26, 27) both at entry to care, and after a diagnosis is subsequently made. Studies report up to 50% of cannabis users that have ED presentations with subsequent hospitalization for cannabis induced psychosis, will go on to develop schizophrenia (28, 29). A broad concern with cannabis use and psychosis is a recent study examining population attributable fractions and incidence of schizophrenia that concluded that first episodes of psychosis would be reduced by 12% if high THC content cannabis was not available (14). It is yet unclear if a similar pathway may exist from ED to development of an anxiety disorder or depressive disorder with cannabis use in youth despite studies of long term cannabis users and cross-sectional survey that show higher rates of these disorders in cannabis users (7, 17, 18).

With the legalization of cannabis in various jurisdictions, there is an emerging focus of research in the understanding of who (e.g., age groups, risk factors) are presenting with cannabis-related adverse mental health events, particularly in an emergency (i.e., ED) situation. The popular point of view that cannabis is relatively harmless to use, which may be increasing ED presentations associated with it, seems to be related to the legal transition from medical cannabis to recreational cannabis permitted use (30, 31). It is evident that ED presentations should be considered as potentially harmful outcomes that need to be examined and considered when discussing the impacts of cannabis legalization. In the absence of a clear understanding of the risk factors for mental health adverse events with cannabis use it can be instructive to examine what cannabis use characteristics are seen with new presentations of mental illness both in EDs.

In this narrative review, we will discuss what is currently known about cannabis-related ED and EMS presentations of mental presentations of mental illness, discussing risk variables and outcomes both prior to and after legalization, including our experiences following cannabis legalization in Canada. We will also discuss what is known about cannabis-related ED adverse events based on gender (including transgender individuals) and biological sex. Where possible, we will discuss the differences between alcohol and cannabis on impact on mental health services to fairly present the magnitude of the impact with the understanding that these two recreational substances may impact different populations of individuals at risk for adverse events.

## Approach To This Review

This is not a systematic review. However, to inform the reader of the approach taken we supply this brief overview of the method. Searches of Pubmed/Medline were conducted from July, 2020 to October, 2020 for the terms cannabis or marijuana and emergency department and adverse events or mental health or prevalence. A similar series of searches were conducted substituting emergency transport, ambulance, emergency mobile units for emergency department. However, the addition of the mental health term to the emergency transport searches was found to be too restrictive so the search was done with emergency transport or ambulance or emergency mobile units (ambulance MeSH terms) and cannabis or marijuana. We did not include presentations due to synthetic cannabinoids in this article. Google scholar was also searched for the same terms. Papers found were then scanned for mentions of mental health impacts associated with confirmed cannabis use in the emergency department and emergency transport setting. The reference lists from the papers located were also hand searched for relevant articles. Published studies from case series to systematic reviews were included in this manuscript.

## Cannabis and ED Presentations for Mental Health Concerns: The Stats We Know

Cannabis-related mental health adverse events precipitating Emergency Department (ED) presentations can include anxiety, suicidal thoughts, psychotic or attenuated psychotic symptoms, and can account for 25–30% of cannabis-related ED visits (32). While these presentations do not constitute a large number of cases overall, they are concerning for the longer term mental health of the presenting individuals. Cannabis-related complaints also account for a small but important proportion of EMS presentations (3.8%) (4). Depending on co-ingestion of alcohol or other substances, 19–37% of these will not be transported to ED as some presentations such as acute anxiety may be managed entirely by paramedics on scene, thus still requiring use of health resources (4). Cannabis-related ED presentations have begun to be explored in more depth recently, due in part to increasing numbers of jurisdictions that have cannabis legalization (medicinal and recreational). In this first section of this review we discuss what we know about the frequency of cannabis-related mental health presentations from a variety of geographic areas.

The literature on this topic is limited in scope, and what does exist is derived mainly from US data. One such example using the Nationwide Emergency Department Sample (NEDS), one of the largest all-payer ED datasets in the US, Shen et al. (33) reported a 7 % increase annually of ED visits associated with cannabis use from 2006 to 2014 (33). While not detailed for diagnoses, they reported that 30% of cannabis use ED presentations were associated with individuals who had a co-morbid mental health disorder. There are additional US studies focusing on state level data. Perhaps not surprisingly, there is a concentration of studies out of Colorado where cannabis was legalized for medical use in November 2012 and recreational use in January 2014. An overall increase in demand for emergent medical care is shown in Colorado state-wide and single site studies that have reported significant increases in ED visits with cannabis-related billing codes for similar time frames (34–36). Wang et al. (37) reported that of those ED visits with cannabis billing codes, mental illness was the most prevalent diagnostic code. Wang et al. (34) also examined an adolescent (>13 and <21 years of age) population in a Colorado tertiary care pediatric hospital system. They reported a statistically significant increase in adolescent cannabis-related ED and urgent cares visits from 2009 to 2015 (34). A Colorado statewide study using a sample size of over 4 million ED visits found a 5-fold greater prevalence of mental health diagnosis among ED visits with cannabis associated codes, compared to ED visits without cannabis associated codes (38). This study used administrative data from the Colorado Hospital Association ED discharge data and looked for a cannabis exposure combined with a mental-health related code as the outcome. It should be noted that the number of cannabis-related ED visits in this data set (0.8%) were dwarfed by the number of alcohol-related visits (36%) (38).

An inner city hospital ED in Flint, Michigan, USA was the site of a prospective study with an online screening survey administered to 14,557 individuals who were admitted to the ED in association with substance use for either medical or injury reasons (39). This survey captured information on substance use (including cannabis) and also used the Short form health survey (SF-12) to gauge a quick measure of the individuals overall physical and mental health. Though not comprehensive, the SF-12 is a good fit for the ED setting where survey time can be limited. In the SF-12's domain of mental health which is characterized as a measure of psychological distress and well-being, substance use was associated with being in the bottom quartile of this measure (40). This study excluded suicidal individuals and while 6% of the sample met criteria for cannabis abuse/dependence, the mental health component was not broken out by substance used (39). Similarly, in Nevada, legalization came into effect for medical cannabis in 2013 and recreational cannabis use in 2017. 40 used the Nevada State ED database and showed cannabis-related ED visits were up 23% from 2009 to 2017. The characteristics of the groups most contributing to this trend were individuals 21 to 29 years old and female sex and 26% of the sample had co-morbid mental health issues (41). Of note, the ages 21–29 demographic comprised 52% of ED visits for cannabis-related complaints in 2017 (41). It should be noted that much of the US data may be underestimating the effects of cannabis as the decision as to whether to go to an ED in the US can depend on medical insurance coverage, as shown by studies showing decreasing appearances by uninsured individuals (42). Interestingly, while it is unknown if this can be generalized outside of the US, there is data suggesting that in a state with legalized cannabis, alcohol is not commonly associated with concurrent cannabis use in either the recreational or medical context (43).

ED usage for mental health concerns after cannabis legalization in Canada has been less well-studied but there is a small body of literature beginning to emerge in this area. Recreational cannabis was legalized for use on October 17, 2018 after cannabis use for medical purposes was regulated from 2001. A crude estimate of morbidity impact in Canada of several cannabis associated events reported an estimate of 106–186 cannabis-attributable incident cases of schizophrenia in Canada per year (44). Most of these individuals will first identify to the ED and while the number is not high, the burden from this chronic condition on a publically funded health care system is measurable. Even prior to legalization the demand for mental health care in conjunction with cannabis use was significant and one study at a small urban center in Ontario, Canada showed that 8% of cannabis-related ED cases required inpatient psychiatric care (45). Hospital admission was more likely for cannabis induced psychosis (45).

The legalization of cannabis for recreational use was in part tied to gatherings in support of the movement on April 20 each year (4, 6–21) so it may be of interest to note that there is a Canadian study examining the impact of cannabis use at mass gatherings of 4–20 celebrations on emergency service demand. A study conducted across 6 regional hospitals in British Columbia, Canada over a 10 year period (2009–2018) showed significant increases in admissions for substance induced mental health disorders and cannabis intoxication on 4–20, compared to control days (46). Studies suggest the need for advance planning for emergency mental health services in conjunction with cannabis mass gatherings.

There have been a small number of studies comparing cannabis outcomes before and after legalization in Canada. One pre-post legalization study found a 45% increase in cannabis related ED visits post-legalization compared to pre-legalization across 14 urban ED centers in Alberta, Canada. Though this is a large percentage increase, this only translated into 3 additional visits per ED per month (47). Interestingly there was a small decrease in visits related to what the authors call psychological co-diagnoses post-legalization, which included psychosis and anxiety related disorders. However, the authors also noted a significant increase in individuals leaving the ED with a cannabis-related complaint without receiving treatment, which may account for the “missing” individuals (47).

Interestingly, there have been reported increases in cannabis-related ED visits in countries where legalization has not occurred, thus reflecting a possible overall societal change in attitudes toward cannabis use. For example, in southern France, Noel et al. (48) reported between 2009 and 2014 a statistically significant increase of ED visits related to cannabis exposure overall and by age group, including rate changes of 12.6 to 24.3/10,000 for 15–20 year olds and 8.0 to 11.7/10,000 for 21–26 year olds. While they reported a higher proportion of males in the 15–26 age group, the F:M ratio in younger age groups was the same (48). In Switzerland, cannabis has been decriminalized for minor possession in 2012 but not legalized. A retrospective study from one center in Switzerland examined all ED visits over a 4 year period from 2012 to 2016 (49). This study found that while <1% of overall ED visits were due to acute illicit drug toxicity, 26% of these cases were related to cannabis, second only to cocaine. Unfortunately, despite mental health effects being reported for the whole dataset they were not divided by specific substances used, which has been a common finding during our literature search for this paper (49). Another large retrospective study from Switzerland was recently published on ED visits related to acute toxicity (50). In the cannabis only group (26% of the sample of 717 visits), the average age was 26, 77% of the sample was male, and 43% of the sample came to the ED by ambulance. Twenty three percentage of these “cannabis only” individuals reported anxiety as their primary symptom. The majority of the cohort was discharged from the ED and considered by the authors as having experienced minor toxicity; however, 7% experienced psychosis and 8% of the cannabis only group were referred to psychiatric care. The most common substance detected in conjunction with cannabis in the other cases studied was alcohol (50). The cannabis and alcohol group presented with more agitation and aggression than the cannabis alone group which had significantly more anxiety than the combination group. Interestingly, there was no difference in the rate of presentations of psychosis between the cannabis only and the cannabis/alcohol groups (50).

In Australia, where cannabis has been decriminalized in some states, one study examined the nursing triage notes of 263 937 ED admission records over a period from 2004 to 2006 from two hospitals in Sydney, Australia (51). Alcohol related presentations far outweighed cannabis ones at 5% for alcohol and 2% for all other illicit drugs combined. Within the 2% of illicit drugs, 14% were cannabis and cannabis had the highest odds ratio (7.6) of being associated with a mental health primary diagnosis code (51). The patients in the alcohol and drug ED visit categories were also more likely to be under 30, and require more ED resources such as arriving by ambulance, being triaged as urgent or be an after-hours visit. This study was interesting also for its design, comparing nursing triage notes to ICD codes, reporting that the nursing free text detected more of the drug related diagnoses (51).

A study from Turkey, where cannabis is illegal except as approved cannabinoid pharmaceutical preparations for medical purposes as per legislation passed in 2016, showed that 44% of ED admissions associated with street drug use were for cannabis (52). However, this only comprised 0.2% of total ED admissions for the urban low income ED under study at a tertiary care center (52). This study reported on the frequency of hallucinations (verbal or auditory); approximately 3% of the sample experienced these psychotic symptoms but the reporting was not categorized by drug used; however, it is worth noting that there were no amphetamines or opioids used by the cohort in this study (52). Again, this study illustrates the challenges on getting broad but detailed data on the impact of cannabis use on mental health.

The literature on the impact of cannabis use on ambulance transport to the ED is very sparse. Despite this, reporting on the existing literature compliments the cannabis related ED presentation studies. The assessment of first responders is the most contemporaneous and well-positioned to capture detailed information about drug use that may be obtained from multiple sources as opposed to the patient themselves. Additionally, we know from the ED studies that a significant number of patients who present with adverse events associated with cannabis use depart the ED either prior to receiving treatment or against doctor's orders. This raises the question as to whether there is another group of patients receiving some EMS care but refusing transport to the ED at all. What we know about cannabis involved EMS attendances is primarily from studies done in Australia. Expanding our focus more broadly to encompass all mental health presentations to the ED, there is some evidence that ambulance transport to the ED is increasing including when substance use is involved, with one study showing an increase from 35.6% in 2004 to 45.1% in 2013 (53). If we look more closely at transport to the ED for cannabis associated events, a review of trends of EMS use over time in Australia showed increasing use of EMS over time, and interesting age-group trends. Patients using cannabis-only tended to be slightly younger (15–29 years of age). Cannabis only individuals also were less likely to be transported to hospital with the non-transportation rate being 37% for this group and an additional 20.7% being assessed as not requiring any further emergency treatment (4). This was significantly different from the cannabis and alcohol combined patients who had the greatest police involvement rate and were more likely to be encountered in public outdoor areas (4). This study also found that rates of cannabis-related ambulance attendances among the total population increased significantly over the study period and concerningly, attendance rates for young females (15–29 years old) associated with only cannabis showed the second highest rate of change in attendances (increasing from only 0.2 attendances per 100,000 population per year to 7.1). Alcohol was by far the most frequent co-intoxicant across the study period (4). One further study out of Norway reported 35% of injection drug overdose EMS contacts were in individuals who used cannabis 2 or more days a week, suggesting a troubling co-use concern (54). Unfortunately, the difficulty in conducting this type of research even in the setting of retrospective database searching is that intoxicated patients will often refuse transport and ambulance crews may not see the value in recording this information so a record of contact is lost (55).

Cannabis use harms are also present in users aged 50 and older. This demographic (ages 45 to 64) is showing significant increases in use levels in Canada post-legalization (3, 56). Cannabis related ED presentations in this population has been found to be associated with greater healthcare usage regardless of amount or frequency of use, and the likelihood of injury was increased with the presence of any mental health disorder in these individuals (57). A study in South Carolina examined what drugs if any were found in the system of patients admitted to the ED who had a pre-existing mental illness and were ultimately admitted into a psychiatric inpatient service from the ED. THC was most common, found in 40% (*n* = 191) of patients with alcohol being third at 15% (*n* = 72) (58). The mean age of this sample was 37 years but ranged from 18 to 97 years (58). Unfortunately, this retrospective study did not breakdown the admissions by mental health diagnosis.

Overall a picture emerges of cannabis-related ED visits with comorbid mental health presentations being not uncommon and may be on the rise. Additionally, while less common than alcohol related ED visits, cannabis-related ED visits may present a higher level of service demand including mental health admissions and follow up.

## Cannabis and Acute Mental Health Presentations

There are fewer studies that have specifically examined cannabis toxicity ED presentations and associated mental health symptoms, and fewer still that directly connect EMS attendances to acute or future mental health symptoms. However, development of acute psychiatric symptoms can be the hallmark of cannabis poisoning or cannabis toxicity. Cannabis poisoning can be considered an accidental overdose resulting in a constellation of physical and mental health side effects, including psychosis, anxiety, and paranoia. When codes for cannabis poisoning were examined in the national emergency department sample in the United States, it was found that individuals who were experiencing cannabis toxicity were significantly more likely to present as having a psychotic, anxiety, mood, or behavioral/emotional disorder and that the association with this presentation was stronger for females than males (59). Shelton et al. (60) employed an administrative database coupled with a chart review for the period of 2012–2016 and found that of cannabis-related ED visits, 24.8% were for psychiatric reasons compared to GI causes at 30.9% and intoxication at 29%. Particularly concerning in this study was that among the acute psychiatric symptoms, 74% of these individuals presented with suicidal ideation, anxiety and psychotic symptoms. They also reported a statistically significant increase in the number of ED visits for each year examined (*p* = 0.016, 0.015, and 0.013 for psychiatric, gastrointestinal, and intoxication, respectively) (60). The Euro-DEN project has studied the acute toxic effects of cannabis. In a study across 10 European countries, 16.2% of ED presentations involved cannabis alone or in combination with alcohol or other illicit drugs. Of the cannabis only presentations that were considered cannabis poisoning/toxicity, the most common mental health presentations were agitation/aggression (22.9%), psychosis (20%), and anxiety (20%). This was not a large sample size (35 cases). However, from a health services demand perspective, it is interesting to note that 21 of the 35 cannabis only cases arrived by ambulance and four were admitted to an inpatient psychiatric unit (32).

New York, USA decriminalized possession of <25 g of cannabis in 1970; however, the law was not uniformly applied so clear legal use was not seen there until legalized medical cannabis use was signed into law in 2014. A 2016 study based on prospective data from two urban hospitals compared 87 patients attending the ED who reported exposure to any cannabinoid to 17 patients who used synthetic cannabinoid receptor agonists (SCRAs) (61). They concluded that SCRAs had significantly greater neurotoxicity than cannabis alone; however, the table of neurological profiles included in the paper shows very similar values between the two patient groups except for agitation which is worse with SCRAs at 41% but still present at 16% for cannabis alone and delirium was only reported for the cannabis group (61). A strength of this study was the confirmation of use within the previous 24 h but a potential weakness is that recruitment only occurred during business hours (61).

Cannabis is often referred to in marketing materials as being an anxiolytic. Though unproven, this assertion is often promoted by staff at cannabis dispensaries (62, 63). This is primarily based on studies in rodents as in humans cannabis is more frequently reported to have anxiogenic effects. There is little evidence to support the anxiolytic properties of cannabis when used by humans. High grade evidence is lacking as shown in a recent meta-analysis and systematic review (64). Acute anxiety can be a feature of cannabis poisoning or acute toxicity. Some naïve users will experience acute anxiety that does not abate quickly and present to the ED. The Nationwide Emergency Department Sample in the United States was used to examine factors associated with acute accidental cannabis poisoning based on ICD-10 codes (59). They found that the association between cannabis poisoning and meeting criteria for an anxiety disorder was significantly higher (adjusted odds ratio of 2.82) as well as criteria for a mood disorder (adjusted odds ratio 2.30) for females than males (59). Measuring anxiety symptom presentations to the emergency department may underestimate the number of cases associated with cannabis poisoning. This conjecture is based on EHS studies, paramedics may often be resolving these presentations without transport (4). This would suggest that acute anxiety presentations may be more prevalent than currently understood but are not sufficiently severe to require ED services. However, this aspect of is not well-studied.

In summary, cannabis use does seem to be directly related to the development of new mental health symptoms in a minority of users but the evidence grade is not high at this juncture and more work is needed.

## Cannabis Use and Emergency Healthcare Service Utilization

The most thorough manner to study ED service utilization with mental health presentations would be to not only collect administrative data but also to attempt to further characterize the patients attending the service. A recent study that is a step toward comprehensively studying the association between cannabis use and emergent service needs prospectively enrolled ED patients with an average age of 45 years (range 18–88 years) who had ever used cannabis. Unfortunately, the majority of participants (60.8%) had not used any cannabis in the past 30 days and it was unclear when the individuals in the group had last used or what their lifetime use pattern was. However, within these limitations the study shows some profound results. The median age for first use was 16 years old. Cannabis motives were examined and the second reason given for use at 30% (*n* = 89) was to treat anxiety and the fourth most common at 17% was to treat depression (65). While this suggests self-medication for individuals enrolled in the study who had mental health conditions, it is important to note that the majority (77%) began using cannabis prior to the onset of their mental health condition (65). Additionally, 59% of patients reported anxiety in the previous 30 days and 46% reported serious depression in the same period. Most concerning, 9% of the sample reported suicidal thoughts in the past 30 days (65). A point in favor of the study, it was a fairly balanced dataset for sex (52% female and 48% male) as well as ethnicity 55% white, 42% African American, and 1.7% Hispanic. A limitation of this study could be considered the lack of clarity around lifetime use levels with the inclusion of those who had ever used cannabis, and similarly, the majority of the sample not having a recent use pattern to compare to outcomes.

The impact of cannabis use on emergency services in conjunction with mental health concerns may be affected by the route of administration. Cannabis edibles can be much more variable with regard to THC content and even exceed the dose delivered by inhalation in some cases (66). While only 0.32% total cannabis sales in Colorado between 2014 and 2016 were edibles, 10.7 % of cannabis-related ED visits were related to edibles (36). Significant levels of intoxication and even accidental coma and death have been reported with cannabis edible use as well as some evidence of increased psychosis risk with intoxication by this route and concerns about lowered age of initiation (67). This underscores the need to further study the various modes of cannabis use to elucidate the strength of these relationships and establish causality.

There have also been studies that focused on examining health care utilization for those individuals presenting with cannabis-related ED presentation. One example is a study examining healthcare utilization by persons with cannabis use disorder in the US using the 2005–2013 National Surveys on Drug Use and Health data that found 40% of their sample reported an ED admission in the past year. The subgroup of individuals who had cannabis use disorder and a major depressive episode in the past year had the second highest prevalence of ED visits at 50% of the group (68). This study also highlights the paucity of studies that examined depressive symptoms and disorders in the context of ED visits associated with cannabis use.

Victims of suicide and suicide attempts will often require EMS and ED services. Suicides are difficult to study in conjunction with cannabis use. Metabolism and circulation cease with death leading to some researchers who study motor vehicle fatalities to contend that THC levels seen in post-mortem samples to be more accurate measure of the amount of THC present at the time of the crash then studies then those sampled in the ED (69). A similar comparison could be made with victims of suicide; however, in both situations victims are not able to self-report cannabis use and the pharmacokinetics of cannabis is such that detectable levels of THC or THC-COOH can be found for 30 days post-last use in daily chronic users (70). This is in part due THC's lipophilic nature and to its resistance to degradation by enzymes used to modulate the endocannabinoid system (71, 72). Thus, it is difficult to make a temporal connection between death and intoxication or direct impact of cannabis in this situation. With this caveat in mind, there are some troubling statistics related to the toxicological detection of cannabis in confirmed suicides. In what is otherwise a review article, Roberts presents data from the Colorado Suicide Data Dashboard showing a 77.5% increase in cannabis positive toxicology for suicide victims pre-post legalization with the caveat that not all suicides had toxicology data available (9). Non-completers with cannabis in their system have also not been well-studied. There is a study using data from a Canadian injury surveillance system electronic Canadian Hospitals Injury Reporting and Prevention Program (eCHIRPP) reporting on 11 pediatric and 6 general emergency departments (ED) across Canada which found that when intent was examined for excessive cannabis use that self-harm was the second most common reason for pediatric cases and third most common for adult ones (73).

The demands of cannabis users on emergency services both ED and EHS are one of the more unmet needs of research on how cannabis impacts healthcare systems and are of pressing importance as more jurisdictions move toward legalization.

## What Happens After Leaving The ED?

As mentioned previously, the Euro-DEN project studied the acute toxic effects of cannabis and though as noted this was not a large sample size (35 cases). What may be most concerning in this study is that 71% of these received no treatment and 86% were discharged/self-discharged (32). While the patient's immediate symptoms may have resolved, it is unclear what the long term outcomes are for these individuals. A limitation of some of the large database administrative studies is the inability to distinguish between unique visits and repeat visits by cannabis users which inhibits the ability to follow a patient's trajectory longitudinally.

There is some collateral cannabis information related to ED admissions for alcohol intoxication in 2006–2007 at a hospital in Switzerland who were followed up regarding their substance use 7 years later (74). While not focused on their cannabis use, this study did find that 7 years after their ED admission, 53% reported past year cannabis use and 87% reported lifetime cannabis use. Men reported significantly more cannabis use but women reported significantly more psychiatric disorders with anxiety disorders being the category leading the difference in the previous 12 months (74). Additionally, 74% remembered the admission that began their enrollment in the study 7 years prior (74).

The situation in Colorado is also interesting from an epidemiological point of view as the past month cannabis use level among native Coloradans has remained constant since recreational legalization but healthcare utilization associated with adverse events due to cannabis has increased (38, 75). Some authors have noted that this may be related to the current market forces being focused on sales with ever increasing concentrations of THC in cannabis products (38). This may suggest a cumulative dose dependency for at least certain types of adverse events associated with cannabis use as has been suggested by others for the development of psychosis (28, 29, 76).

## Where Do We Go From Here?

It may be useful to contemplate what the emergency department primarily administrative data is suggesting for longer term implications for mental health. This is unfortunately a thought exercise as even less literature focuses on what the care pathway, if any, may be for individuals who present to the ED with a cannabis-related mental health issue especially if it is an index mental health presentation. It should be stressed that these presentations do not constitute a large number of cases. The majority of particularly occasional cannabis users will not experience these types of adverse events. However, the number of cases requiring intensive emergency care resources such as transport by ambulance and inpatient psychiatric care indicates that these minority of cases can be healthcare resource intensive. The presence of conditions such as cannabis induced psychosis should constitute a public health concern. While we are slowly seeing a growing body of literature for the impact of cannabis-induced psychosis on repeat ED visits, for other mental health conditions, such as anxiety disorder, we do not know very much about the frequency of repeat ED visits and the degree to which they are relying on a revolving door of ED services to fill a mental health service gap (28, 29). Given that we know that a significant percentage of individuals who experience a psychotic episode will go on to develop a psychotic disorder, a routine referral from the ED to psychiatric care to monitor the individual post-ED would seem a reasonable approach to consider. It is not clear if anxiety symptoms severe enough to warrant emergency care will eventually actuate into an anxiety disorder in a similar continuum to what is postulated for cannabis and psychosis. The situation in the literature is similarly lacking when one examines major depressive disorder or bipolar depression. The literature regarding ED outcomes with cannabis use specific to these populations is very limited. Though there is at least one report of major depression as an adverse event with medical cannabis use which is not the focus here (77). The lack of research in these areas is not surprising given the challenges of doing research in urgent care and across disciplines to obtain outcomes for longer term psychiatric care. This lack of information further impacts clinical care as if we knew the frequency of conversion from a severe adverse mental health event related to anxiety symptoms or depressive symptoms with cannabis use to a diagnosed disorder requiring ongoing care, clinical guidelines could be developed. As we move to greater cannabis use with greater acceptance of the product, the ED may be one of the sentinel locations to monitor any emerging mental health trends.

There are also opportunities for public education that may be possible in the ED setting. The effects we present here are, we suspect, more commonly associated with higher (often defined as 12% and greater) THC concentration strains of cannabis with little to no cannabidiol in the material as these are the most commonly sold strains in the marketplace in legalized settings (78, 79). The sale of these higher THC strains is based on consumer preference (80). However, there is evidence that consumers do not understand the significance of the percentages of THC and CBD in sales materials in the legal marketplace (81). As this research moves forward, some differentiation between strains of cannabis and the relative content of two of the most common cannabinoids in the plant by weight should probably be part of the discussion. Individuals who use recreationally and who have an adverse event may not be aware that their choice of strain may have impacted their medical outcome. Additionally, the popularity of edibles and their use by youth to help conceal use for a variety of reasons should be addressed as this formulation of cannabis is disproportionately associated with adverse events (82). However, it is not clear how well-understood the risks of using edibles are by youth or how well strain differences are understood among consumers overall.

There are limitations to the literature cited in this review that need to be considered when we try to move forward with research in this field. A number of the studies cited here mention being hampered by inconsistently applied ICD-9 or ICD-10 codes when examining administrative databases. Additionally, some studies mention being unable to distinguish between unique and repeat visits (51). Some of the prospective studies were only conducted during business hours which does not coincide with the known profiles of greatest demand for services related to cannabis users (50, 83). Lack of cannabis strain information but also lack of route of administration data hamper our ability to translate ED findings into public health education materials on the risk of various forms of cannabis use.

Another common limitation in this field of study is illustrated by many of the ED studies that are focused on psychosis and psychotic symptoms as an outcome. An example of this type of study is a published abstract from Alberta, Canada specifically examined substance induced psychosis at one urban ED and examined the presentation and outcomes for these cases. The study was not large at 44 cases but had an interesting case presentation as they were more likely 15–20 years old (35%), experiencing persecutory delusions (65%), and unlikely to be experiencing isolated visual hallucinations (9%) or to have a previously diagnosed psychiatric condition (32.5%). These patients were admitted to inpatient psychiatric services and the average length of stay was 6 days (84). The study infers that if admitted in an emergent context, it is possible that complete resolution of symptoms will occur in these substance induced presentations. Indeed, the literature is consistent with acute use of cannabis inducing self-limiting psychotic episodes that are reflected in the rate at which individuals are released from the ED without treatment. However, like many of the papers that we found in this field, when patients are classified as “substance using” frequently this refers to all mind altering (also called illicit) substances lumped as a group. So while there is nothing wrong *per se* with this study, it is an example of a potential missed opportunity to parse the impact of cannabis use as it is not isolated from other substance use as a group. The work to examine the association with cannabis misuse is most clear in psychosis but anxiety disorders and depressive disorders are also potentially impacted by regular use and more study is needed in the emergency context.

An additional facet of the impacts of cannabis use and mental illness that could not be discussed here as we found very little directly addressing this issue, is the increased use in pregnancy and decrease in perceptions of cannabis harms for pregnant women (85). It is unclear if pregnant individuals are presenting to the emergency department with mental health concerns as none of the data presented here recorded the pregnancy status of the women presenting to the ED despite data showing that pregnant women with a history of depression and anxiety are more likely to use cannabis during pregnancy (86). The only conditions that we could find literature for were an association between cannabis use during pregnancy and preterm birth which conceivably could require emergency services and a suggestion of cannabis use during pregnancy leading to increased nausea and vomiting also potentially requiring emergency intervention (87, 88). Additionally, in the longer term, the reported increases in levels of cannabis use during pregnancy may also lead to increasing numbers of individuals who were exposed *in utero* with behavioral outcomes that may be associated with a further cycle of cannabis harms that end in ED use [reviewed in (89)].

A final point to consider is how we could comprehensively arrange the data to enable larger epidemiological studies with more depth. There is a mechanism for reporting adverse events from cannabis use to the FDA in the United States (90) and the Government of Canada through Health Canada runs a website for reporting cannabis recalls, and adverse reactions (91). These systems may also be a mechanism to track the prevalence of adverse mental health events associated with cannabis use (92). This may be especially important in a setting where mental health impacts of cannabis are not generally captured in the usual hospital injury databases (93). However, harmonization of the data collected would be required.

## Author Contributions

CC wrote the first draft. All other authors contributed edits and comments to the first draft. CC and PT prepared the final draft. All authors approved the submission.

## Conflict of Interest

The authors declare that the research was conducted in the absence of any commercial or financial relationships that could be construed as a potential conflict of interest.
